# Indigenous Maya-Mam leadership competencies: a grounded theory study

**DOI:** 10.3389/fsoc.2024.1463562

**Published:** 2025-01-10

**Authors:** Pablo Lamino, Amy E. Boren-Alpízar, Jason Headrick, Scott Burris, Carlos Carpio

**Affiliations:** ^1^Department of Agricultural Education and Communication, University of Florida, Gainesville, FL, United States; ^2^Department of Agricultural Education and Communications, Texas Tech University, Lubbock, TX, United States; ^3^Agricultural Economics and Agricultural Business Department, New Mexico State University, Las Cruces, NM, United States

**Keywords:** Indigenous leadership, leadership competencies, Guatemala, Indigenous studies, resilience

## Abstract

**Introduction:**

This study explores the leadership competencies within the Indigenous Maya-Mam community, aiming to understand the specific skills and qualities exhibited by Maya-Mam leaders. The research seeks to address the gap in literature regarding Indigenous leadership practices, particularly focusing on how cultural values influence leadership behaviors.

**Methods:**

Qualitative methods were employed for this study, including interviews and thematic analysis. Data collection took place in various Maya-Mam communities in Guatemala, where participants were selected based on their roles and experiences within the community leadership structures.

**Results:**

The study identified several key competencies among Maya-Mam leaders, including leading by example, promoting inclusive leadership, valuing bilingual proficiency, and emphasizing community solidarity. These competencies underscored the leaders’ roles as both inspirations and facilitators of community development.

**Discussion:**

The findings suggest that Maya-Mam leadership is deeply rooted in cultural values and community dynamics, influencing how leaders engage with their roles and responsibilities. This aligns with broader research on Indigenous leadership, highlighting similarities and unique aspects of Maya-Mam leadership practices. The study underscores the importance of understanding local contexts and cultural values in leadership development initiatives within Indigenous communities. Future research could further explore comparative studies across different Indigenous groups to enhance understanding and inform effective leadership strategies.

## Introduction

Indigenous leadership has been explored from various perspectives, including psychosocial, organizational/sociological, and community-centered viewpoints (Hofstede, 2001; Salmon et al., 2022). Each of these perspectives provides valuable insights into the dynamics and practices of leadership. Psychosocial theories focus on individual traits, motivations, and interpersonal relationships, offering a framework for understanding how personal qualities influence the connection between Indigenous leaders and their followers (Hofstede, 2001; Gambrell, 2017). Organizational and sociological theories, in contrast, examine leadership within the broader group dynamics, addressing how leaders navigate traditional values in the face of evolving social and economic systems (House et al., 2004; Kriger and Seng, 2005). On the other hand, community-centered perspectives view leadership as rooted in social relationships and collective goals, closely aligned with Indigenous values such as reciprocity, communal responsibility, and cultural preservation (Hartog et al., 1999; Minthorn and Heather, 2019).

These theoretical approaches provide a rich foundation for understanding leadership within Indigenous communities, whose cultural, historical, and social contexts have shaped their identities and ways of life (Julien et al., 2010; Castillo, 2010; Wesley-Esquimaux and Calliou, 2010). Among these communities, the Mayas are well-known for their rich legacy, deep-rooted traditions, and remarkable leadership qualities. The Mayas are mainly situated in Mexico, Guatemala, Belize, Honduras, and El Salvador (Minority Rights Group, 2021) and have played a crucial role in their communities by conscientiously safeguarding their cultural heritage while navigating the intricacies of contemporary society (Maya Educational Foundation, 2023).

In Guatemala, Indigenous peoples comprise 43.8% of the national population, with 41.7% self-identifying as Maya (National Institute of Statistics of Guatemala, 2019; International Labor Office, 2021). In certain departments such as Totonicapán, Sololá, and Alta Verapaz, the Maya population constitutes the majority, accounting for 98, 96.4, and 93% of the total inhabitants, respectively (National Institute of Statistics of Guatemala, 2019; Xiap Riscajché et al., 2014). Unfortunately, poverty and inequality are prevalent issues in these areas, with Indigenous peoples being disproportionately affected (Bogin, 2021; Ippolito et al., 2017). These disparities in social and economic outcomes are further exacerbated by the country’s heightened vulnerability to the adverse effects of climate change (Central Intelligence Agency [CIA], 2023).

Within the 22 Maya cultural groups (International Work Group for Indigenous Affairs [IWGIA], 2023), the Mams hold a unique position. The Mams are primarily located in the departments of Huehuetenango, San Marcos, and Quetzaltenango (Wills, 2022). With their traditional clothing, intricate textile weaving, and oral storytelling traditions, they possess a distinctive cultural identity that sets them apart (Ciborowski et al., 2021). The Mam language, which belongs to the Mayan language family, symbolizes their heritage and unity (England, 2003; Joshua Project, 2016). Despite facing challenges such as poverty, limited access to resources, and marginalization, the Mams demonstrate resilience and commitment to preserving their cultural identity and advancing their communities (Ciborowski et al., 2021; Wills, 2022).

In terms of leadership, the Mams are highly valued within the Indigenous community of the region (Gardner and Richards, 2017). They have established unique traditions and structures aligning with their values and community dynamics (Hernandez et al., 2022). Mams leaders are responsible for guiding their communities, settling disputes, and advocating for their interests in external situations (Gardner and Richards, 2017; Hernandez et al., 2022). The leadership qualities of the Mams are shaped by the wisdom and knowledge passed down through generations, which enables them to successfully navigate the intricacies of contemporary society while preserving their cultural identity (Ekern, 2011; Caxaj et al., 2014).

In recent times, there has been a growing interest in examining leadership beyond the conventional theories established in the US and Europe (Abeyta et al., 2020). This interest also extends to Mesoamerican territories, which possess a significant representation of Indigenous people (Hall and Patrinos, 2014). Experts in the field of Indigenous Leadership emphasize the importance of researching Indigenous groups within their cultural contexts (Zhang et al., 2012). In this regard, the exploration of leadership abilities demonstrated by Indigenous Mayas, such as the Mams, has become a crucial task. While conventional leadership theories prove useful in various scenarios, they often fail to capture the true essence of Indigenous leadership and the exceptional qualities that Indigenous communities bring forward (Caxaj and Kolol Qnan Tx'otx' Parroquia de San Miguel Ixtahuacan, 2018). Therefore, exploring Indigenous Mayas’ leadership competencies with a culturally sensitive and context-specific perspective is important to expand the study of leadership.

To bridge the existing knowledge gap, a qualitative grounded theory research approach was employed to better understand Mam Indigenous leadership. The study explored the perspectives and experiences of two Maya-Mam communities in San Marcos and Huehuetenango to enhance our understanding of Indigenous leadership competencies within this unique context. The knowledge gleaned from this research will be valuable for future interventions and studies supporting Indigenous leadership development. Ultimately, increased comprehension of Mams’ leadership practices and competencies can pave the way for informed strategies and initiatives that empower Indigenous communities and foster their leadership potential. This study aimed to develop a conceptual framework for Maya-Mam Indigenous leadership competencies. The research was guided by the question: How do the Maya-Mam conceptualize Indigenous leadership competencies within their community?

### Leadership competencies

This study adopted a leadership competency model as its conceptual framework, a model that has been a subject of debate regarding its contextualization. Different researchers, including Chouhan and Srivastava (2014), Megahed (2018), and Shippmann et al. (2000), have provided diverse opinions on defining leadership competencies, considering attributes such as aptitudes, underlying characteristics, abilities, or observable skills.

As the understanding of competency has evolved, researchers have begun to consider multiple variables in its definition, including a combination of skills, abilities, and personal characteristics, as well as knowledge, understanding, qualities, attributes, values, beliefs, and attitudes (Dubois et al., 2004; Page et al., 1994).

**Figure 1:**
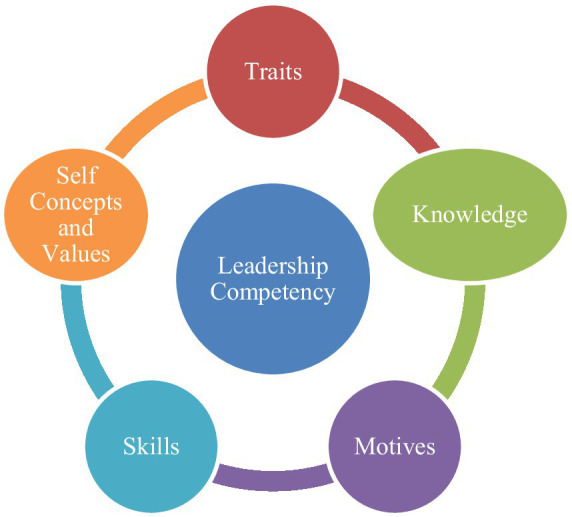
Leadership competencies (adapted from Spencer and Spencer, 1993).

This research is based on Spencer and Spencer’s (1993, p. 12) definition of competency as the “underlying characteristics of an individual that are causally related to effective or superior performance in a job or situation.” According to Spencer and Spencer’s (1993) competency model, there are five key components in all competency definitions: motives, traits, self-concept and values, knowledge, and skills. Motives refer to a person’s underlying drivers for specific actions or goals, traits encompass physical characteristics and consistent responses, self-concept and values involve attitudes and self-image, knowledge pertains to information and learning, and skills represent task performance ability (Čukanović-Karavidić et al., 2016; Spencer and Spencer, 1993). Figure 1 shows the leadership competencies.

## Materials and methods

### Design

The study utilized a constructivist grounded theory design (Charmaz, 2006) due to the scarcity of existing research on Maya-Mam’s Indigenous Leadership. Grounded theory methodology involves an iterative process of data collection and analysis, ensuring the use of accurate data to develop a theory (Charmaz, 2006; Corbin and Strauss, 2015). This approach employs constant comparative analysis, synthesizing diverse concepts to form a coherent theoretical model that emerges from the data (Corbin and Strauss, 2015; Glaser and Strauss, 1967). Additionally, the study adopted a social constructivist philosophical paradigm to interpret the perspectives of the Maya-Mam community and its leadership (Creswell and Creswell, 2018).

The study obtained approval from the Texas Tech University Institutional Review Board [IRB] under the assigned number IRB2022-518. Participant safety was prioritized, with measures in place to ensure confidentiality and anonymity during data collection and analysis. To accommodate illiterate participants, informed verbal consent was obtained before conducting interviews, as this method allowed for easier comprehension and participation. This study qualified for exemption.

### Setting

**Figure 2:**
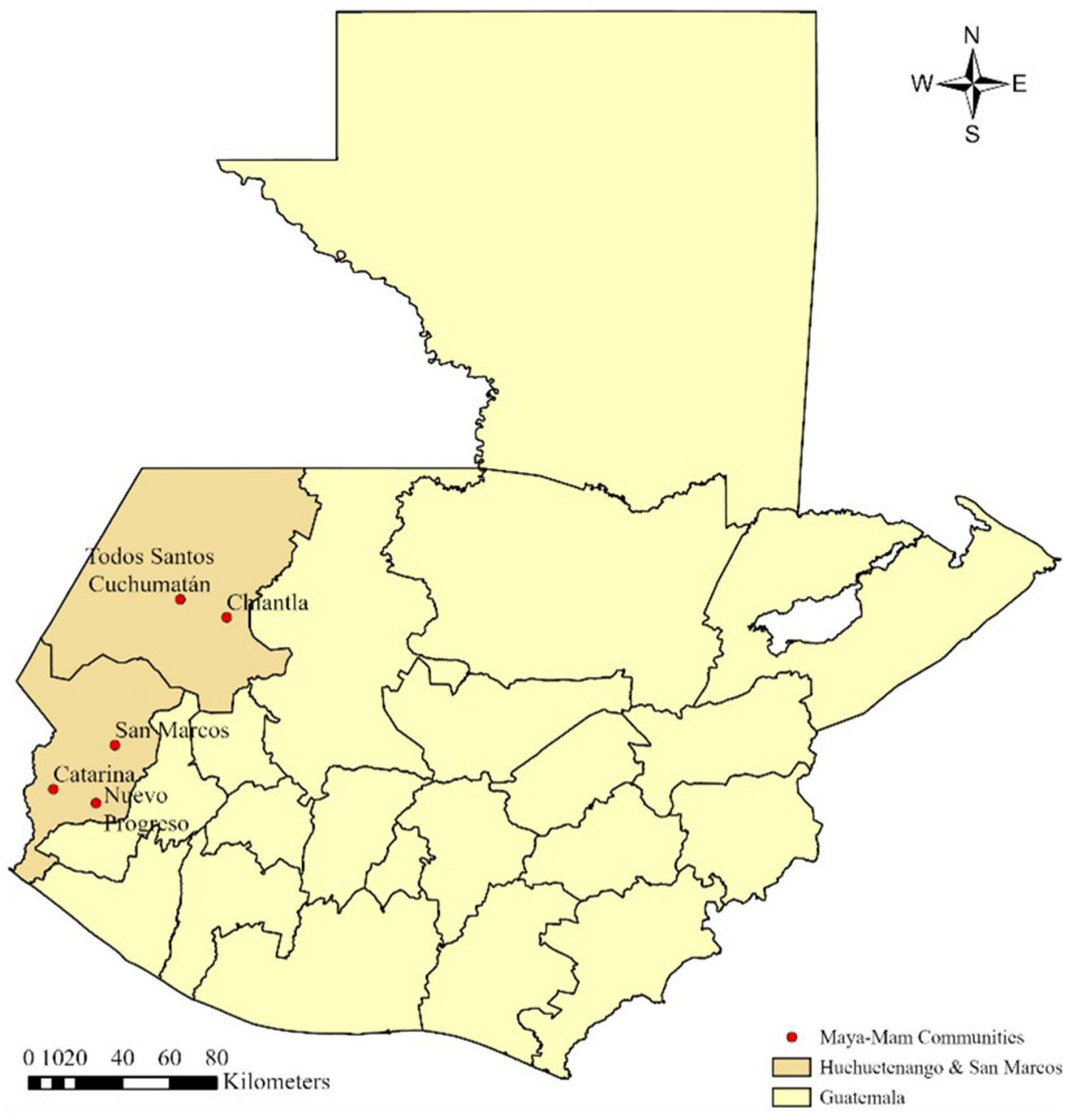
Maya-Mam communities visited.

The study was conducted in the Guatemalan departments of San Marcos and Huehuetenango, where the first author and principal investigator [PI] resided for 15 days in a Maya-Mam community in Cuchumatanes, Huehuetenango, and 15 days in a Maya-Mam community in Santa Catarina, San Marcos, to learn and record Mam cultural dynamics (Charmaz, 2006). Additionally, PI organized meetings with Mam leaders and followers to build trust and conduct interviews. Figure 2 presents the location of the communities visited during the study.

### Participants selection

Purposeful sampling was used to collect meaningful insights from individuals with first-hand knowledge of the Indigenous leadership process (Aurini et al., 2021; Charmaz, 2006; Patton, 1990). Purposeful sampling involves deliberately selecting cases that meet predetermined conditions (Creswell and Creswell, 2018).

For this research, the following conditions were considered. First, participants needed to have direct involvement with a Mam community. Second, they had to self-identify as Mam. Third, they were required to possess the capability to discuss the Indigenous leadership process. Finally, affiliation with the community was an important criterion. Participants under the age of 18 were excluded from the study.

Recruitment primarily relied on collaborations with non-profit organizations active in the targeted communities. Additionally, the principal investigator (PI) engaged directly with these communities, enabling him to identify individuals who had served in various leadership positions or were currently in follower roles. In this study, a “leadership position” was defined as any formal or informal role in which individuals influenced or organized community efforts, represented the community in external matters, or led initiatives of communal significance (Rost, 1993; Yukl, 2012). Such roles include community elders, local council representatives, coordinators of cultural or agricultural programs, and organizers of social gatherings. Participants identifying as followers were those actively involved in community activities under the direction or influence of these leaders (Kellerman, 2007; Chaleff, 2009).

The study included 49 participants, with 28 individuals from Cuchumatanes, Huehuetenango, and 21 from Santa Catarina, San Marcos. For clarity, Table 1 describes the participants from Huehuetenango, including their age, gender, and community. Similarly, Table 2 presents the corresponding characteristics of the participants from San Marcos.

**Table 1:** Description of Huehuetenango participants (*N* = 28).

Pseudonym	Age	Sex	Community
1. Tatiana	54	Female	Nuevo Paraíso
2. Beatriz	49	Female	Nuevo Paraíso
3. Tomasa	35	Female	Nuevo Paraíso
4. Carmen	47	Female	Cuatro Caminos
5. Sofia	47	Female	Cuatro Caminos
6. Hector	40	Male	Libertad
7. Gina	40	Female	Libertad
8. Rosario	38	Female	Libertad
9. Natalia	20	Female	El Rosaio
10. Ovelia	18	Female	Cuatro Caminos
11. Yasury	21	Female	Aldea Chavez
12. Waleska	19	Female	Aldea Chavez
13. Rodolfo	25	Male	Cuatro Caminos
14. Oscar	56	Male	Cuatro Caminos
15. Darvin	25	Male	Cuatro Caminos
16. Sara	34	Female	Cuatro Caminos
17. Andrea	22	Female	Nuevo Paraíso
18. Clementina	42	Female	Nuevo Paraíso
19. Alejandro	61	Male	Chiantla
20. Jose	36	Male	Cuatro Caminos
21. Mirna	25	Female	Aldea San Pablo
22. Jennifer	28	Female	Cuatro Caminos
23. Carla	24	Female	Cuatro Caminos
24. Berta	22	Female	Cuatro Caminos
25. Luciana	28	Female	Cuatro Caminos
26. Norman	18	Male	Todos Santos
27. Maria	23	Female	Nuevo Paraíso
28. Eunice	57	Female	Todos Santos

Pseudonyms were used for each participant to protect their confidentiality.

**Table 2:** Description of San Marcos participants (*N* = 20).

Pseudonym	Age	Sex	Community
1. Bernardo	37	Male	San Marcos
2. Paola	26	Female	San Marcos
3. Norma	54	Female	Nuevo Progreso
4. Rocio	58	Male	San Marcos
5. Israel	54	Male	San Marcos
6. Anastasia	45	Female	Blanca Flor
7. Rodolfo	69	Female	Blanca Flor
8. Leon	59	Male	Blanca Flor
9. Antonio	24	Male	Nuevo Porvenir
10. Saul	58	Male	Nuevo Porvenir
11. Flor	49	Female	Nuevo Porvenir
12. Fabiana	20	Male	Nuevo Porvenir
13. Clara	18	Female	Nuevo Porvenir
14. Emperatriz	53	Female	Nuevo Porvenir
15. Roberto	52	Male	Blanca Flor
16. Celeste	35	Female	Blanca Flor
17. Amapola	35	Female	Blanca Flor
18. Lorenza	35	Female	Nuevo Porvenir
19. Julieta	24	Female	Nuevo Porvenir
20. Anabel	25	Male	San Marcos
21. Elena	33	Female	San Marcos

Pseudonyms were used for each participant to protect their confidentiality.

### Data collection

Various methods were employed to collect data, including face-to-face in-depth interviews, researcher memos, participatory and non-participatory observations, and photos (Aurini et al., 2021; Charmaz, 2006; Creswell and Creswell, 2018).

### Interviews

To gather information on Maya-Mam Indigenous leadership competencies, a total of 49 one-on-one semi-structured interviews were conducted. While research questions guided the interviews, they allowed flexibility for new ideas and themes to emerge, and additional questions were incorporated based on the nature of each interview (Erlandson et al., 1993).

After each interview and during significant moments, PI used “memoing” by jotting down inquiries, comments, personal feelings, and reflections. This practice helped capture important information that could not be recorded (Saldaña, 2013).

### Observations

Qualitative research relies on observation as a valuable approach to studying participants in their natural settings (Schensul et al., 1999). Therefore, two types of observation were employed in this research: participant observation and non-participant observation. In participant observation, the researcher takes an active role in the activities being studied to gain a better understanding of the context and participants’ viewpoints. In contrast, non-participant observation involves the researcher observing from an external perspective without direct involvement (Aurini et al., 2021; Laurier, 2010).

During the semi-structured interviews, non-participant observation techniques were utilized (Laurier, 2010). Participant observation techniques were employed during communitarian meetings meant to socialize potential communitarian projects. PI focused on observing interactions, listening attentively, and taking detailed field notes (Spradley, 2016). Before the observations, participants were informed about the purpose and process, allowing them to ask questions (Laurier, 2010).

### Photos

Photography served as a complementary method to gain insight into the participants’ daily lives and Indigenous culture. The photographs supplemented the data collected through interviews and observations, providing a visual dimension to the research (Pain, 2012). To ensure participants’ confidentiality, PI obtained verbal consent for photograph usage and assured them that their faces would be blurred if the photos were used. Visual data enriched the analysis and deepened the understanding of participants’ everyday experiences (Balmer et al., 2015; Barbour, 2008).

### Data analysis

The data analysis followed the grounded theory approach, incorporating theoretical sensitivity, theoretical sampling, coding, memoing, and sorting, utilizing the constant-comparison method (Creswell and Creswell, 2018; Kolb, 2012). The constant comparative analysis was used throughout the coding process to ensure robust meanings of codes and categories. This involved comparing data within and between documents to identify conceptual similarities or differences (Glaser and Strauss, 1967; Kolb, 2012). Conceptually related data were grouped into themes, while data that did not align were left for further exploration during axial coding.

The constant comparative analysis allowed PI to identify and analyze similarities and differences in participants’ stories and determine relevant, robust, and meaningful codes that did not fit the existing theory. Despite its complexity and challenges, constant comparative analysis was crucial in developing an emergent theory.

Additionally, open coding, axial coding, and selective coding procedures were applied, constantly comparing the data within the study (Aurini et al., 2021). Open coding involves identifying and labeling emerging concepts, categories, and themes without preconceived ideas. Axial coding organized the codes from open coding into related groups and established connections between them. This process structured the data into a coding paradigm, forming a theoretical model. Finally, selective coding involves carefully selecting core categories, establishing connections, verifying relationships, and refining and developing categories as needed.

### Trustworthiness

Ensuring the trustworthiness of qualitative research, especially when using a grounded theory approach, is crucial (Lincoln and Guba, 1985). To enhance this study’s rigor, measures were taken to address credibility, transferability, dependability, and confirmability (Creswell and Creswell, 2018).

Credibility, accuracy, and truthfulness of the data and interpretations were ensured through participant engagement, diverse observation techniques, and data triangulation from multiple sources (Cope, 2014; Creswell and Creswell, 2018). Transferability, akin to validity in quantitative research, was addressed by purposive sampling and rich, detailed descriptions of the findings (Cope, 2014; Lincoln and Guba, 1985). Purposive sampling selected participants based on specific characteristics, while detailed descriptions facilitated the application of findings in other contexts.

Dependability, similar to reliability in quantitative research, was achieved through transparent reporting of data collection and research procedures (Lincoln and Guba, 1985). Data were stored in a dataset, including interview transcriptions, reflexive memos, and official documents. A researcher’s journal documented challenges and essential information encountered during field research.

Confirmability, eliminating bias and ensuring consistency, was addressed through research reflexivity and inter-rater reliability (Creswell and Creswell, 2018). Research reflexivity involved the researcher’s awareness and transparency regarding their background and potential biases. Inter-rater reliability ensured consistent conclusions among different researchers analyzing the same data. Documentation of the research process and double coding, where two or more researchers independently analyzed the same data set and reconciled their interpretations, significantly enhanced confirmability (Creswell and Creswell, 2018).

## Results

**Figure 3:**
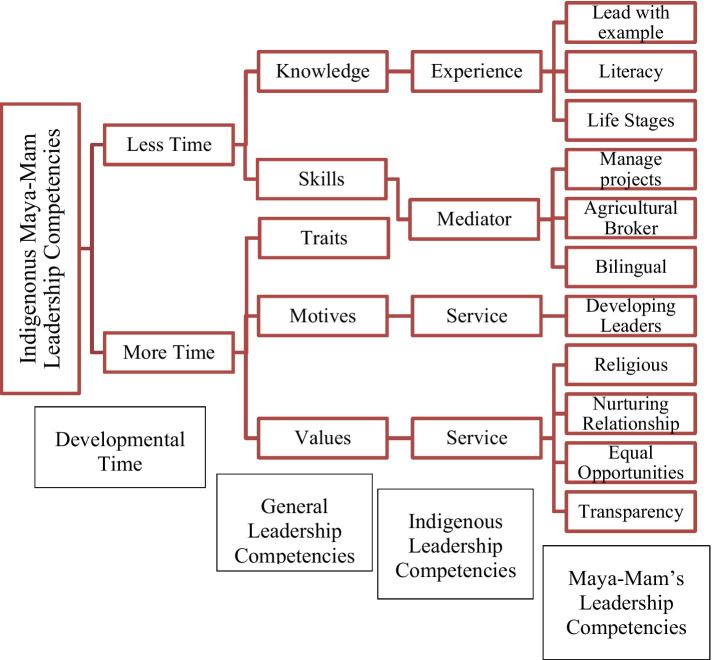
Indigenous Maya-Mam leadership competencies.

The initial research inquiry aimed to identify and elucidate the essential competencies possessed by Indigenous leaders. Through data collection and analysis, a theory emerged that delineated the Maya-Mam Indigenous leadership abilities. The findings of this research are presented by integrating them into a comprehensive framework and providing a detailed explanation for each component. Figure 3 illustrates the Indigenous Maya-Mam leadership competencies [IM-MLC].

### Developmental time

When building competencies, it is important to consider the Developmental Time component. Different competencies require varying amounts of time to develop and can be categorized into long and short periods. For example, knowledge and skill competencies generally require less time to develop as they can be improved through training and experience. However, competencies related to traits, motives, and values often require more time because they involve personal change that is acquired across life stages (Chouhan and Srivastava, 2014; Megahed, 2018; Spencer and Spencer, 1993).

Developing skills and knowledge is a measurable process that can be monitored and improved through structured training and experience. In contrast, developing traits, motives, and values is a more complex and time-consuming journey as these qualities are deeply personal and individual. Therefore, self-reflection, self-awareness, and deliberate efforts are necessary to cultivate and align with one’s values and aspirations (Čukanović-Karavidić et al., 2016; Spencer and Spencer, 1993).

### Leadership competency: knowledge

Knowledge acquisition is essential to a person’s understanding and comprehension of a specific subject or domain. It can be obtained through various means, such as formal education, self-study, research, and continuous learning (Lin, 2019; Spencer and Spencer, 1993). In the case of Maya-Mam leaders, their Indigenous knowledge leadership competency focuses on conceptual and practical knowledge. They learn about principles and apply them to real-life situations. Their Indigenous knowledge is often based on direct experience, similar to other Indigenous groups (Bruchac, 2014). However, their formal education is limited to high school-level knowledge (International Labor Office, 2021).

### Indigenous competency: experience

In Indigenous communities, leaders are expected to have a range of leadership experience, and elders are highly respected for their knowledge and expertise (Bruchac, 2014; Ekern, 2011; International Labor Office, 2021). This theme was analyzed in various contexts, identifying three Maya-Mam leadership competencies: (1) leading by example, (2) literacy, and (3) life stages.

### Maya-Mam Indigenous competency: leading by example

This sub-competency came to light as interviewees acknowledged instances where past leaders resorted to demagoguery to persuade people that they were the right candidate, only to behave differently once in power. For instance, Israel, a 54-year-old resident of San Marcos, emphasized this phenomenon during his interview. He said:

A leader is someone with principles, honor, and honesty. A leader also makes effective not only what he says but also shows that he is a leader with his actions. That is a true leader, not the charlatans who talk too much and do nothing for us.

Participants highlighted the rigorous evaluation and scrutiny faced by leaders from the public and fellow leaders within the community. Beatriz, a 49-year-old resident of Huehuetenango, pointed out that leaders are continuously observed and judged based on their everyday actions. She expressed: “A leader must oversee the community. He can work well, or he can work badly. And if he works badly, the other leaders call him on it so that they can work well to improve the community’s wellbeing.”

Amapola, a 35-year-old resident of San Marcos, distinguished between two types of leadership within Mam communities: positive and negative. Positive leadership leads to beneficial outcomes, while negative leadership involves actions that harm the community. Her reflections underscore the significance of different leadership styles on community well-being. Amapola shared:

There is a positive and negative leader. So, a positive leader is a person who works for the community’s common good. The negative leader is the authoritarian who says, “Things are done as I said.” So, the leader must be open; he must be the one who organizes, not the one who decides alone.

Maya-Mam community members evaluate potential leaders based not only on their family management but also on their past leadership experiences and contributions to the community. Darvin, a 25-year-old resident of Huehuetenango, highlighted that leaders are chosen based on their successful project implementations in previous medium-level positions like secretary or treasurer. He said:

Firstly, they [leaders] already have experience; they worked on programs. Secondly, since they are, let us say, they are also exemplary and have previously carried out projects in other leadership positions. So sometimes they are elected again to be leader, or if they [the community] do not like them, they change the leader.

**Figure 4:**
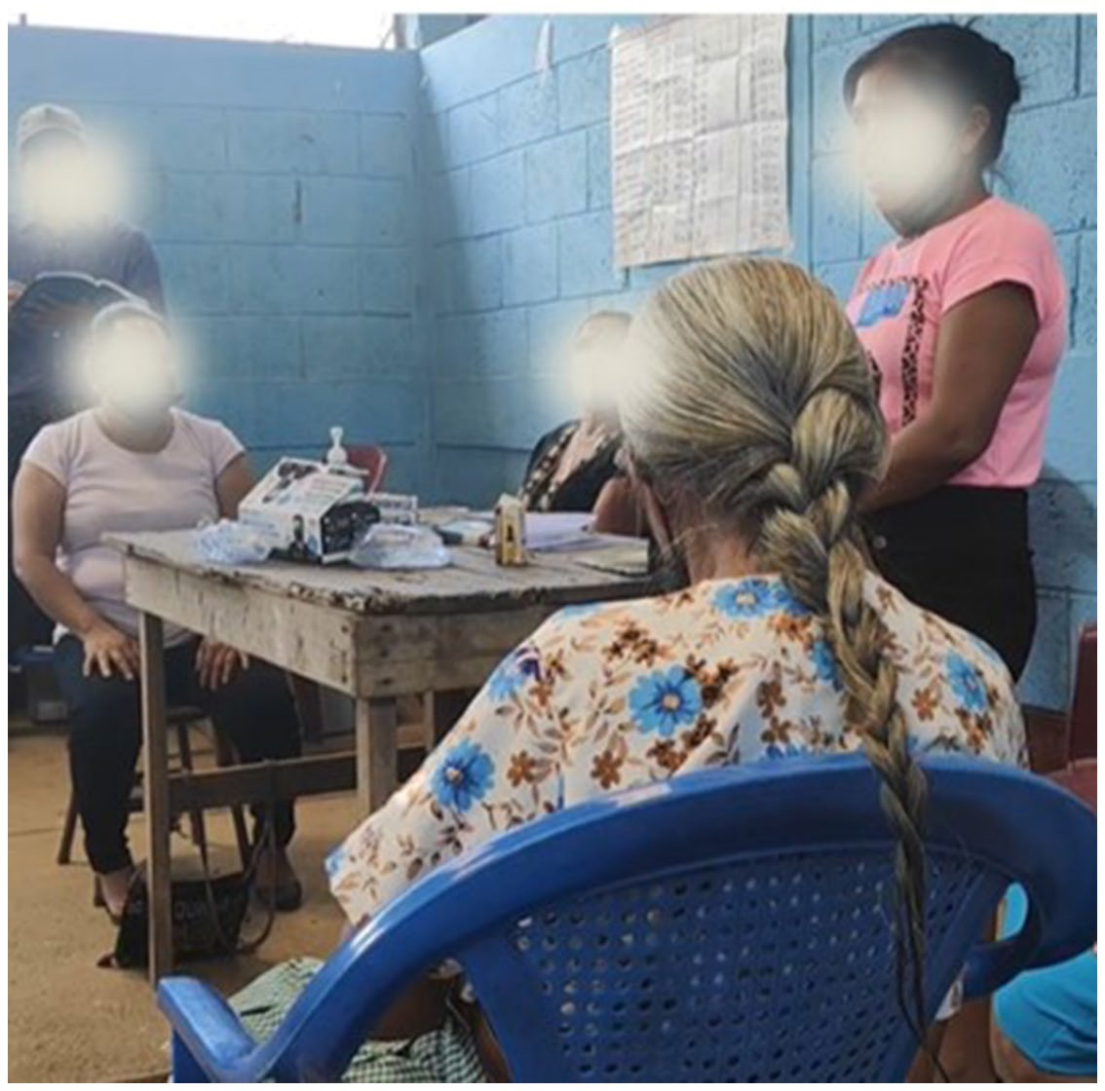
Example of a community secretary.

During field observations, PI documented community events, using photography as visual evidence. PI observed the positive effects of developmental programs for women, fostering empowerment and connectivity. Many women were inspired to pursue leadership roles, starting with positions like secretaries or community witnesses, considered medium-rank leadership roles. In Figure 4, the communitarian secretary is seen reading previous meeting minutes, illustrating community involvement.

### Literacy

Within the Maya-Mam community, there is a strong emphasis on the ability to read and write for individuals who aspire to leadership positions (International Labor Office, 2021). While no official mandate requires this, the belief is that possessing reading and writing skills contributes to effective leadership. This perspective highlights the importance placed on educational achievement within the community, even though it is only focused on literacy.

During interviews with the Mams’ community members of Huehuetenango, it was generally agreed that completing a high school education is a sufficient qualification for a leadership role. Clementina, aged 42 from Huehuetenango, echoed this sentiment, stating that those who have completed high school possess the required intellectual abilities to be considered for leadership positions. She expressed: “We selected the leader because he is a little more developed, for example, in his studies, he has a little higher school education.”

Saul, a 58-year-old from Huehuetenango, agreed with Clementina’s comment that their leaders were selected based on their high school education, which equipped them with the necessary skills to read, write, and lead projects. He stated:

We chose him because we believed that he could guide us with his ability. Because not everyone can, but we prefer people who have completed high school. He [leader] knows how to read a little or knows how to write. He can talk to outsiders to manage a project better, that is why we chose him.

Although some comments indicated that rural communities value competencies beyond literacy, many participants still reported being unable to participate due to illiteracy. Carla, a 24-year-old from Huehuetenango, shared her experience: “What happens is that I do not know how to read, and people who cannot read cannot serve the community.”

In line with Carla’s point of view, Sofia, a 47-year-old Huehuetenango resident, acknowledged that her limited knowledge and inability to read and write may impede her from taking on a leadership position. She said: “No, because we [illiterate people] do not have that much knowledge, right? Since I could not study, my parents did not allow me to study or attend school. Therefore, I do not know how to read or write.”

Carmen, a 47-year-old resident of Huehuetenango, shared her perspective on feeling inadequate for a leadership position due to her illiteracy, despite having valuable experiential knowledge. Through PI research memos and field notes, he discovered that participants may experience internal barriers, such as self-perception and self-doubt, which can limit their confidence in taking up leadership roles. Furthermore, PI highlighted that some individuals prioritize literacy as a criterion for leadership, even if their experiential knowledge could be valuable in serving the community.

During the interviews, it was pointed out that the problem of illiteracy harms potential leaders who have worked hard for their communities but lack formal titles. An example was given by Ovelia, an 18-year-old resident of Huehuetenango, who talked about a midwife who provides essential services to women in the community. Unfortunately, due to her illiteracy, the midwife refrains from seeking a leadership role. She stated: “The truth is, I would like to, but she does not. She [the midwife] says no because the truth is that she cannot read. Well, then, she does not dare.”

### Life stages

During discussions about leadership in Mam’s communities, the participants emphasized the importance of age in selecting future leaders. The perception of age as a significant determinant in the leadership process reflects the community’s cultural values and traditional practices, where wisdom and experience are typically associated with older individuals.

Initially, it was noted that attaining legal age, which is 18 years old, grants individuals’ full access to the decision-making process. Ovelia shared her experience of being able to vote once she turned 18, stating that those who are 18 years old and over can cast their votes. She mentioned: “Yes, those who are already over 18 years old can vote.”

Expanding on Ovelia’s remark, Antonio emphasized the importance of age in Mam’s society. Specifically, Antonio noted that individuals are granted fresh possibilities upon turning 18, regardless of their gender. He stated: “In general, all the 18-year-olds there begin to vote for everything. Yes, everything, be it a woman or a man.”

According to the interviewees, an important age group is when community members reach 25–26 years old, as they can take on middle leadership positions like secretary, treasurer, or community witness. However, they may start even earlier if they are married and have a family. Antonio shared his thoughts on how having a wife can influence one’s ability to take on a middle leadership position at the age of 25. He shared:

As for the young people [assuming a leadership role], if they already have their wives, their women, then yes, they can be elected, but if any young person that is 20 to 25 years old is not [married], they are not involved, because they are single. Only if they already have their women, then yes, they are considered.

According to the interviewees, the third category pertains to individuals who reach the age of 50 and are eligible for a higher leadership position, such as that of a community president. Additionally, leaders who turn 60 have the option to either continue in their current position or give others a chance to lead. Celeste shared her thoughts on these age-based criteria. She stated:

Young people no. They must be 50 years old and up. Young people are as councilors, secretaries there are also two cases, as secretary and there they can be young people who have studies as well. The 50 years are coming [They will be 50 years old soon enough], where the community and its people also give to them so that they obtain a position of power. 60 years pass, if they want to participate, you talk to them and see if they are still willing to work, and if not, they will be the ones who are already reaching 50.

Tatiana mentioned that one reason for choosing leaders who are 50 years old is that often, older leaders pass away, requiring the need to rebuild leadership positions. In her words: “Why did they choose him? Because perhaps many had already passed away, those who were older and had good conduct from living in the community chose him, but later there was no one else, and some began to die and then they chose him because he was older.”

**Figure 5:**
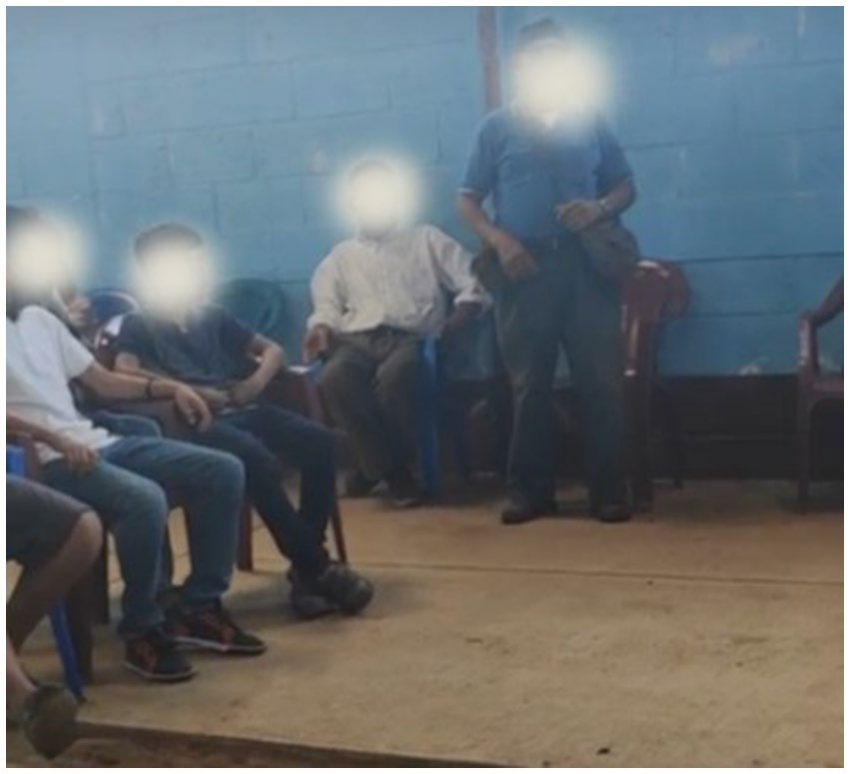
An elder community leader opening the meeting.

On PI trips to San Marcos, he observed that the older members of the community held prominent leadership roles. Upon interviewing the participants, they revealed that this system has been in place for years and is trusted by community members due to positive outcomes. In Figure 5, Saul, the community president of Nuevo Porvenir, can be seen leading a meeting.

### Leadership competencies: skills and traits

Skills encompass specific abilities and proficiencies that individuals acquire to perform tasks or activities effectively. Skills can be enhanced through deliberate practice, targeted learning, and practical experience (Moser-Mercer, 2008; Spencer and Spencer, 1993).

Maya-Mam leaders possess diverse interpersonal and transferable skills that are integral to their leadership roles within their communities. They work closely with individuals and demonstrate exceptional abilities in addressing challenges, exhibiting effective communication, and problem-solving. As mediators and agricultural brokers, they play a crucial role in bridging the gap between their community and the larger world by conveying and translating information and involving their followers in the decision-making process.

Traits refer to inherent personal qualities and characteristics that shape how individuals think, behave, and interact with others. Examples of traits include adaptability, resilience, empathy, and integrity. While traits may have a genetic or innate component, they can also be developed and strengthened through self-awareness, feedback, and intentional effort (Sadiković et al., 2019; Spencer and Spencer, 1993).

Maya-Mam leaders rely on a combination of personality, behavioral, and emotional traits that greatly contribute to their efficacy as leaders. These traits enable them to communicate effectively, respond appropriately in diverse situations, and cultivate empathy towards their followers. By possessing traits associated with effective communication, cultural brokerage, and shared leadership, Maya-Mam mediators can exert influence and inspire others.

### Indigenous competency: mediator

The Mediator leadership competency theme emerged from the data analysis since Maya-Mam perceived the leader as the individual in charge of connecting the community with the external world, like other Indigenous groups worldwide (Cherro Osorio and Best, 2015; Press, 1969). Participants anticipated that it not only represents the cultural and linguistic bridge; it implies the importance of leading external projects for community improvement. Therefore, participants described the mediator into three Maya-Mam leadership competencies: (1) project management, (2) agricultural broker, and (3) bilingual.

### Maya-Mam Indigenous competency: project management

According to the interviewees, Mams’ leaders are expected to possess strong project management skills, which are considered crucial. These leaders play a vital role in bridging the gap between the community and project initiatives. Their authority is significant as any proposed project seeking implementation in the community requires their approval first.

Andrea, a 22-year-old resident of Huehuetenango, highlighted the necessity of the leader’s approval for any program that aims to be introduced. She expressed: “Yes, if a project comes, you must talk to him.” In response to Andrea’s comment, Bernardo, a 37-year-old resident of San Marcos, stated that any government action requires the leader’s approval before implementation. He mentioned: “Any act that comes from the government, outside, from the capital, cannot be carried out without the leader.”

Communitarian leaders have the responsibility of managing local projects as well as seeking out programs outside of the community (Cherro Osorio and Best, 2015). Saul explained that leaders are tasked with finding external programs and establishing relationships with the mayor and other community members to secure donations that will aid in the community’s efforts. In Saul’s own words:

They[leaders] go, they get out of here. They are going to manage a project with the mayor, they are going to talk to the mayor. So, we cooperate one, two, three, or five quetzales for your trip. In this case, here is a car that belongs to the community, and we only help them to buy some gasoline, because they are going to talk to the mayor on behalf of us.

Following Saul’s comment, Rodolfo, a 25-year-old from Huehuetenango, stated that the community fully supports their leader and has provided all necessary resources to effectively communicate their needs to the municipality. He explained: “Look, if the leader is good at managing a project, we all need to help the community and help him [leader] serve the community, and therefore we help him carry out some projects or errands that the leader needs and help him.”

**Figure 6:**
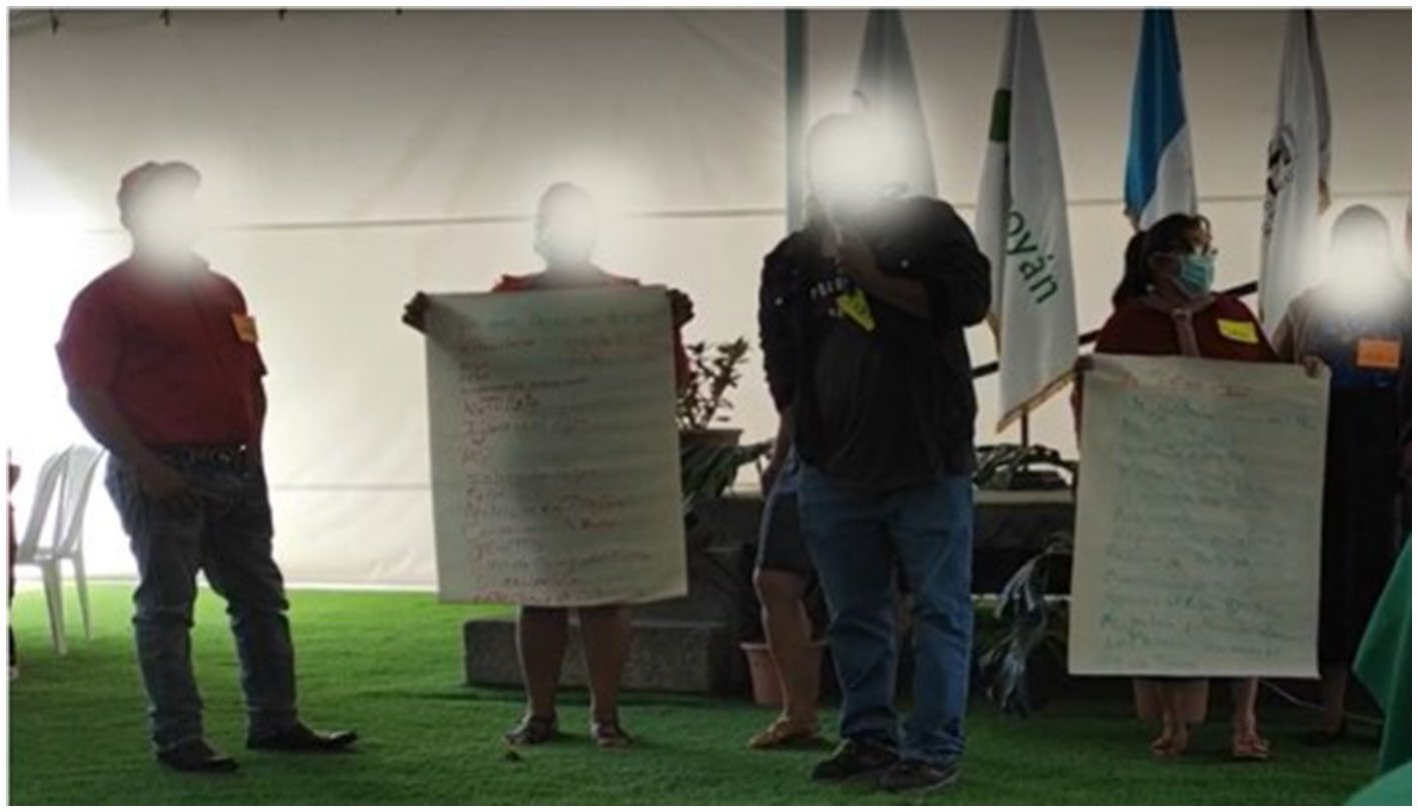
Members of San Marcos community requesting assistance from a non-profit organization.

In the PI’s role as a researcher, He was fortunate enough to attend many community events and program meetings. One gathering that stands out in my memory was hosted by an agricultural non-profit organization. During this meeting, he witnessed a community seeking further support and discussing the next steps in their collaboration with the organization. In Figure 6, a photo taken at the meeting shows members of the San Marcos community eagerly requesting assistance from the non-profit.

Interviewees emphasized the significance of having a community leader who prioritizes and initiates projects to enhance the community’s welfare. Leon, a resident of San Marcos aged 59, cited an example of a former leader who negotiated better payment for community members, thereby improving their lifestyle and reducing the scarcities they faced. He stated:

For example, we had a leader who was managing to get paid better. We, on the farms, would go to work for 20 quetzales [2.57 dollars], I have seen a lot of suffering in my life. I suffered because I started to earn 20 quetzales. It was about 300 quetzales [38.52 dollars] for the year, and I had my family and my wife. And with that, I did not have [anything]. But when we saw what the leader was managing. He was a good leader.

### Maya-Mam Indigenous competency: agri-cultural broker

In rural areas, agriculture is a major source of employment and livelihood (International Fund for Agricultural Development [IFAD], 2011). Participants emphasized that community leaders are responsible for ensuring farmers receive fair prices for their agricultural products. In San Marcos, where coffee production is prevalent, securing better prices from buyers is an essential skill for leaders. By negotiating favorable terms, leaders can enhance community members’ economic well-being in agriculture.

Leon emphasized the significance of having a trustworthy individual who values and safeguards their agricultural produce. He pointed out that before they arrived in the community, the area was overrun with weeds. He said: “We all look at how when we entered here, the coffee was not there, as it is now. Coffee was not like how it is now. Because when we walked in here, this whole area was muddy. It was abandoned.”

Bernardo and Mirna, a 25-year-old woman from Huehuetenango, believe that community leaders should ensure proper agricultural commercialization. Bernardo stated that the leader is accountable for purchasing and selling coffee. He emphasized the importance of this responsibility. Mirna expressed that community leaders must guarantee good agricultural commercialization. Bernardo clarified that the leader is responsible for buying and selling the coffee. He shared: “The leader is in charge of the purchase and sale of the coffee.” Mirna stated that one of the responsibilities of a leader is to collaborate with farmers in the production of their agricultural goods. She explained: “Let us say, they have work, and they go to work with farmers to harvest cauliflowers, fumigate, and all that.”

During the interview, Bernardo highlighted the crucial role of coffee production in the community. He specifically pointed out the leader’s responsibility in selling and converting the coffee, which involves the transformation of harvested coffee cherries into marketable coffee beans. He explained:

As coffee farmers, our focus is on the process of converting coffee cherries into parchment coffee. Unfortunately, some leaders have failed in this process due to poor drying methods or theft, which ultimately reduces profits for the coffee farmer. However, our leader has done an excellent job in providing a good conversion and ensuring a fair profit for the coffee growers.

Flor emphasized that it is the responsibility of leaders to provide coffee improvement workshops to residents and collaborate with other leaders to create opportunities that address their needs for enhancing the commercialization process of coffee. This can include initiatives like improving the roads. She stated:

I do not know how you could call them who work helping with the coffee, they go directly to the coffee crop [extension workers], and they come to evaluate it. They come to give training as well as to take care of the coffee plantation. So that is the job of the leader, the coffee, and the community, they are the ones who promote going to the mayor if there is any need in the community.

Participants expect leaders to take responsibility for converting and selling coffee at a good price. Israel, a former community representative, noted the overwhelming nature of this responsibility. He expressed:

It is hard because under that responsibility falls everything that is maybe territorial problems, and administration problems. When is the harvest? Well, under the president, he is in the coffee drying process. So that’s a bit of a difficult [leadership] charge. But having people who are collaborative and hardworking. I did well in those years as the leader. I’m glad to know that [everything] turned out well.

Participants mentioned that they received praise for improving the quality of their coffee and being open to changing their agricultural practices. Amapola shared that the previous leader’s effective actions inspired him to extend help to other communities in improving their agricultural production. She explained:

I admire [the leader]. He lives in El Cedro, but he has his plot here, but he does not live in the center. I believe he lives in San Marcos because he is serving several communities, so he travels to all the communities where we are from his community. He is the person who manages projects. More than anything on the land, the agrarian problems.

**Figure 7:**
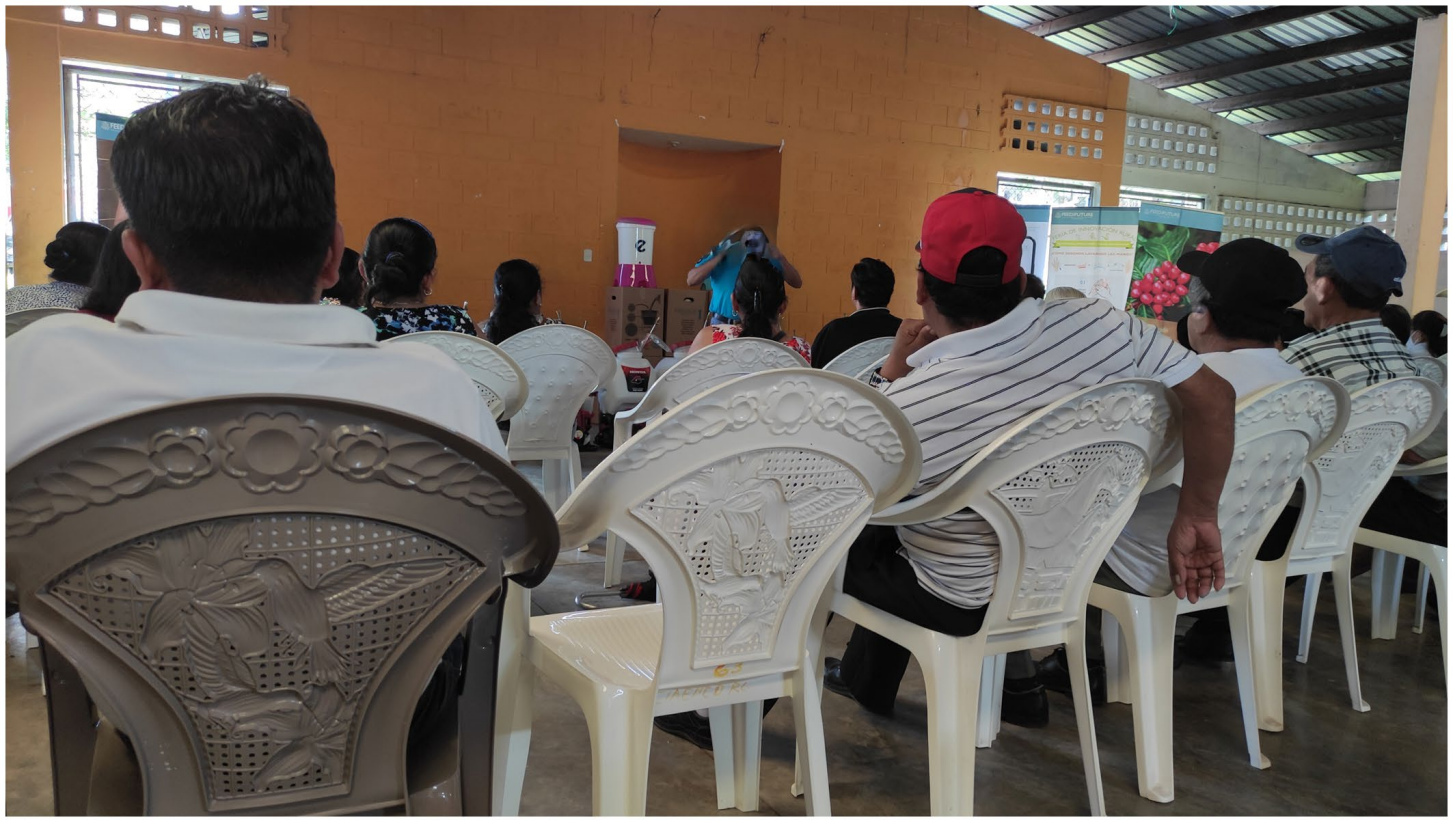
Participants attending a meeting focused on new agricultural innovations for the community.

During a visit to a Maya-Mam community, PI observed a community leader advocating for new agricultural innovations, including water eco-filters and pest-control bombs. The leader noted that due to pollution in their springs, the community needed alternative ways to purify their water and also sought support for pest control. Figure 7 illustrates how the leader communicated with community members about the new advancements we brought in to improve agricultural production.

### Maya-Mam Indigenous competency: bilingual

Bilingual competency is indeed a highly valued skill expected from Indigenous leaders within the Mams community (International Labor Office, 2021). One of the primary reasons for this expectation is the need for leaders who can effectively communicate the community’s needs in Spanish, the dominant language in wider society. The Mams people are known for their Mam language, which may pose challenges in accessing resources and services due to language barriers (Ciborowski et al., 2021).

The significance of bilingual competency becomes even more pronounced for Mams residing in Huehuetenango, as their location further distances them from the main city (International Labor Office, 2021). The limited availability of extension workers who can communicate in both Mam and Spanish exacerbates the difficulties in implementing developmental programs. The language barrier can hinder effective communication, coordination, and access to essential resources and services for the community (Ciborowski et al., 2021).

By possessing bilingual skills, Indigenous leaders from the Mams community can act as crucial intermediaries, bridging the gap between the community and external entities (Ciborowski et al., 2021). Bilingual competency becomes a vital factor in overcoming the challenges posed by the language barrier and promoting the community’s well-being and development (Cherro Osorio and Best, 2015).

Beatriz stated that when choosing a leader, they seek someone who can communicate proficiently in both Mam and Spanish. Additionally, they value someone who is not only intelligent but also willing to share information. She emphasized this as an important trait.

Yes, they were [leader 1] and [leader 2]. Yes, they were very intelligent. They served in anything related to the health post. They speak both languages, and some people can only speak Mam. Then she says the other in the Mam. Among all, they voted for them because they speak both languages.

According to Darvin, effective communication with the municipality requires a basic understanding of Spanish. Therefore, they are seeking a leader who possesses such skills. Darvin emphasized that this is an important factor: “They can talk with others, with the mayor, they do projects. They speak a little Spanish and Mam. They also have good, good, good behavior.”

When PI visited a Mam community in Cuchumatanes, Huehuetenango, he encountered communication challenges with the locals. PI struggled to convey my message using words. Fortunately, a field technician who spoke Mam fluently helped with the translation. This experience highlighted the importance of having bilingual leaders in Indigenous communities.

Natalia reinforced PI observation by stating that for projects or resources to be effective in the community, it is necessary to involve the community in the translation of all information. She specifically mentioned: “Sometimes when assistance or projects come, what he [the leader] does is get the people together and explain it to them in Mam. He spends the whole time saying what actions need to be taken and telling it to the people.”

### Leadership competency: motives

Motives refer to the reasons why individuals behave and make decisions, based on either psychological or physiological factors (Rodgers and Loitz, 2009). In the case of Maya-Mam leaders, social and achievement motives are crucial in identifying and grooming new leaders for the advancement of the group. Furthermore, these leaders often exhibit power motives, which enable them to exert influence and make a positive impact on the group.

### Indigenous leadership competency: service

The description of service competency is consistent with other research on Mesoamerican Indigenous leadership, which states that leaders are elected to ‘lead by obeying’ (referred to as “Mandar Obedeciendo” in Spanish) in study by Casado and Gómez (2010). In the case of Maya-Mam leaders, their main service motive is developing leaders that could replace them in the future.

### Maya-Mam Indigenous competency: developing leaders

Participants highlighted the importance of developing leadership skills among the younger generation. They recognized that investing in the new generation’s development would prepare them for future leadership roles once the current elders step down. This underscores the need for a continuous cycle of leadership within the community.

Furthermore, the participants shared how leaders have encouraged and guided their followers to develop their leadership competencies. This involved providing mentorship, guidance, and growth opportunities. By investing in the development of leadership skills among community members, leaders can ensure a sustainable leadership pipeline and a smooth transition of leadership responsibilities.

Julieta, a 24-year-old resident of San Marcos, shared that when choosing their leaders, they prioritize qualities such as effective leadership skills, clarity in their work, and a dedication to supporting the community’s values. Furthermore, Julieta stressed the importance of selecting leaders who actively encourage and uplift female community members. She expressed:

First, seeing their leadership capacity, there is the clarity that they can have to lead the community, because the idea is to work with the entire community to defend their qualities and that they make themselves available during the hours of the job. I want the leader to be a compañera [female comrade] too. [Women] are willing to give their time and more. I support the compañeras. That is what we want, that our compañeras are also in those spaces.

During a conversation about developing leadership within the community, I inquired about the skills and qualities that are expected from leaders. The participants expressed that while not everyone possesses natural leadership abilities, it can be developed through open discussions on the topic. Elena also acknowledged that a common limitation in leadership is a lack of understanding among people. She said:

Well, leadership is something that not everyone considers because not everyone has the opportunity. So those of us who can, [we] believe that leadership is when we have a space to talk because sometimes people do not understand what leadership is. Many do not understand. They do not participate in a group; they do not understand what leadership is.

Anabel, a 25-year-old woman from San Marcos, acknowledges the importance of having a platform to discuss leadership. However, she notes that some individuals may not be willing to engage in such conversations or collaborate with the community to cultivate leadership skills. She explained:

Look at the qualities. They seek it based on their development and their intelligence because not all of us have the intention to do something, to work with our community. Not all people; actually, there are very few. So, they see the person who seeks development, and who likes to serve the community.

During the data collection process, participants offered valuable suggestions on how Mams’ leaders could enhance their leadership skills. One recurring suggestion was the continued implementation of developmental programs, such as the Saving and Internal Lending Communities [SILCs] workshop.

**Figure 8:**
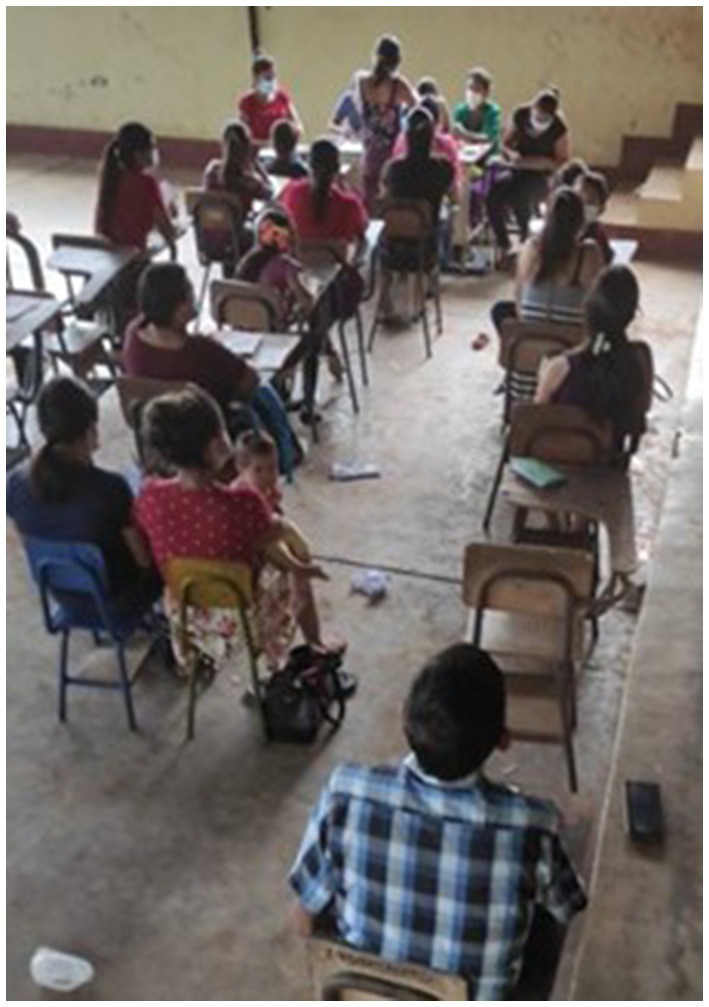
Participants replicating SILCs.

As PI conducted field observations and captured visual evidence through photos, it became evident that programs like SILCs presented opportunities for developing leadership among community members. In Figure 8, it can be observed participants actively replicating SILCs, fostering increased participation and engagement among the entire community. Anabel supported the idea of leadership responsibility, of developing leadership through this kind of program by mentioning the community’s improvements. She stated: “Well, leadership I think is what they are doing. At least come to teach the people here to have an idea of how to get ahead.”

**Figure 9:**
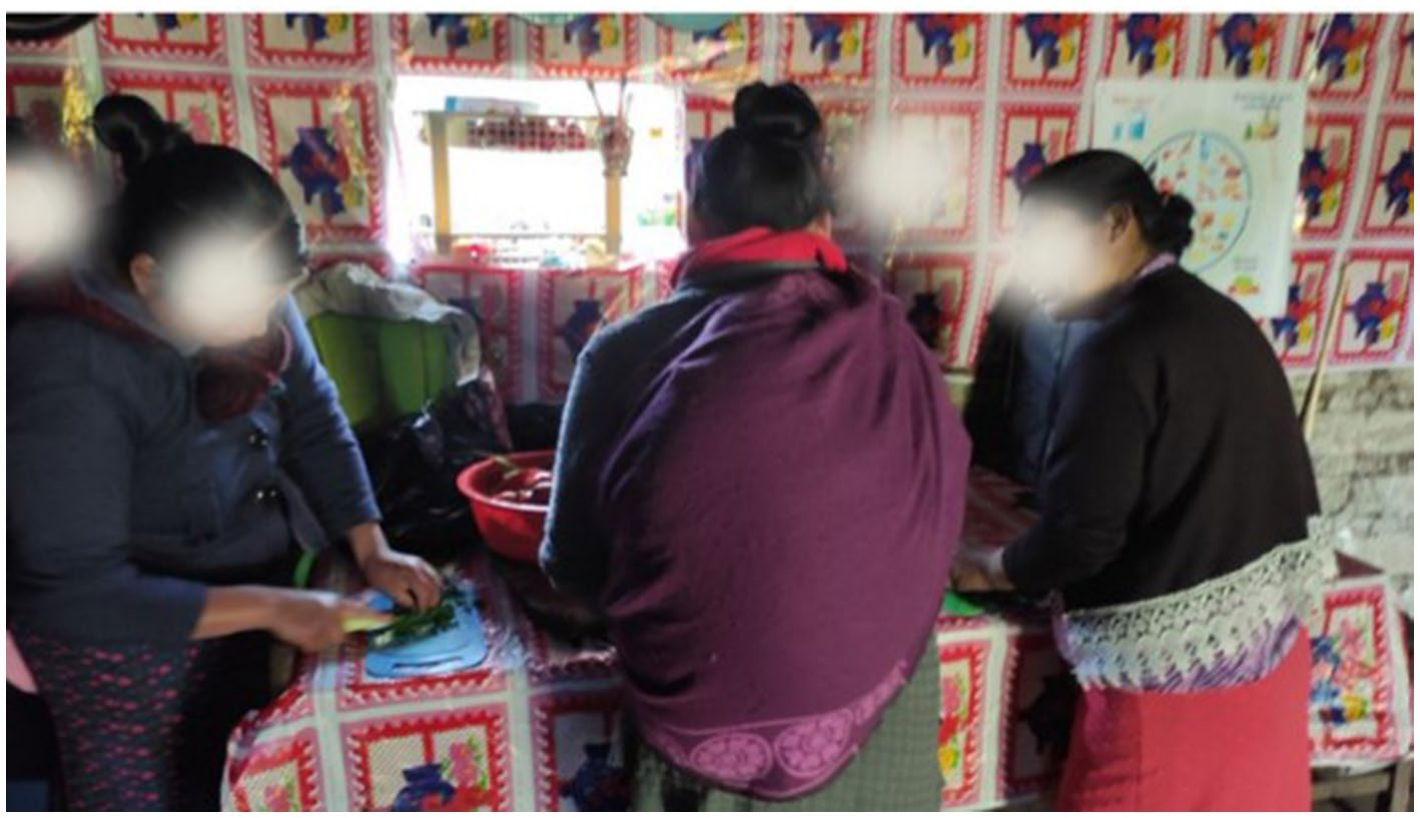
Participants replicating the mean preparation process with other members of the community.

During PI field observations, he actively documented the activities taking place within the community, including capturing visual evidence through photographs. One notable observation was the training received by one of the leaders on the preparation of healthy food, which they were now sharing with other community members. Rosario, a 38-year-old woman from Huehuetenango, shared this information during our conversation. She mentioned: “We were actively participating and learning how to prepare meals from our leader.” In Figure 9, it can be seen the participants replicating the meal preparation process.

Ovelia also emphasized the significance of developmental programs in creating leaders who can train other leaders. She shared an inspiring story about a World Vision participant who chose to become a midwife and assist women during labor or when faced with health issues. She claimed: “But one day was when she went out to a meeting. When she came, she said that she was going to be a mother guide [midwife] for a World Vision program, and from there she began to include others.”

During the interviews, participants were asked if they would be interested in becoming a community leader. They responded positively, expressing their desire to learn more and contribute to the community through teaching. Elena, one of the participants, shared that she would gladly accept the role as she believes it would allow her to motivate and support people, thereby aiding in the development of the community. She said: “Yes, I would accept it. I like the development of my community. I like to encourage people to do a project for their benefit.”

Norma was unsure if she would accept; however, she recognized the opportunities. She expressed: “Maybe I would accept because there everything is learning [I could learn so much there].” Tatiana was uncertain about taking on the role of leader, but she acknowledged the significance of consulting with elders beforehand. This is a customary practice that is anticipated in the community. She mentioned: “I suppose, maybe yes, but I would have to train myself with some who are already old leaders, I would have to tell them, well, I would like to know what the position is, what position I can take, what I can decide with the community.”

During a conversation, Lorenza, a 35-year-old resident of San Marcos, shared her thoughts on how training programs led by former leaders have made women more aware of the significance of receiving training and their essential role in the community. She claimed:

What happens is that we have already received a little training, we are already aware that things are learned, and one is not born knowing. But many women are afraid and say, “I can,” or “I cannot right now, but I will be able to [in the future].” If they are still on the lookout, I’m going to ask for permission to see if they want to or not.

As the leader, Flor recognized a limitation in the development programs and the critiques she received from other women. She expressed disappointment that some organizations committed to attending events but failed to show up, causing frustration. She expressed:

Women’s organizations have always come like this. But what happens is that sometimes we get busy taking care of things more at home and sometimes we promise them some things too. They tell me, “Look, get organized, such a project will come.” And in the end, it does not come. That is why the women say no. Also, if there is no support for women, then it is better, better that there is nothing, she says.

Clementina emphasized the significance of family and recognized that sometimes, they are unable to attend workshops due to the lack of approval from their husbands. This poses a challenge for the leadership. The attendees expect their leaders, especially their wives, to encourage women’s participation. Moreover, they indicated that once the leaders allow women to participate, it also encourages children, regardless of gender, to join. Clementina explained:

You can see that when we sometimes get together in groups, my companions talk to each other, well, right now, well, first, our husbands have set us free. On the other hand, now we do not want to be in the leadership position, because we are giving our children the opportunity, whether male or female, they have the same rights, so they go to study, and I think that’s how it’s going to change.

During PI observations, he noticed that in cases where male leaders are unable to attend meetings, they often ask their wives to act as their representatives instead of delegating the responsibility to the vice president of the community. Saul confirmed this observation by stating that leaders often lack the time to attend meetings and therefore prefer to have their wives take charge instead of assigning the task to the deputy leader. Saul expressed his thoughts as follows: “Because sometimes the leader does not have time to go to the meeting, sometimes the leader’s wife goes, everyone, the women, is given the right to say what they think, what they feel.”

### Leadership competency: values

Values are beliefs and principles that people and societies consider important. They are shaped by personal experiences, family background, and cultural and societal influences (Kesberg and Keller, 2018; Spencer and Spencer, 1993). Maya-Mam leaders are expected to prioritize community interests and support group goals while upholding personal and cultural values. They also inherit cultural conservation values from previous generations.

### Indigenous leadership competency: service

Indigenous leaders have a value-based service competency rooted in their experiences and cultural heritage. This competency is driven by their desire to promote the well-being and advancement of their community (Kesberg and Keller, 2018; Spencer and Spencer, 1993). Their deep-rooted values form the foundation of their commitment to serving their community in a way that aligns with principles of inclusivity, respect for traditions, community welfare, and sustainability. They make decisions with these values to actively work towards improving their community (Xiap Riscajché et al., 2014). Participants described the value-based service into four sub-competencies, (1) religious, (2) nurturing relationship, (3) inclusion, and (4) transparency.

### Maya-Mam Indigenous competency: religious

Religious value competency emerged since they expect leaders to be connected to the church and serve in that capacity emerged from the data, as participants highlighted the value of religious leadership and its impact on the community. According to their perspectives, a good leader actively serves and is involved in the church, as they play a significant role in sharing knowledge about religion.

Participants recognized the influence of religious leaders in imparting moral values, guiding community members, and fostering a sense of unity and spirituality. By being actively engaged in religious activities, leaders not only provide spiritual guidance but also contribute to the overall well-being and cohesion of the community.

They stated that their involvement in the church has helped them enhance their leadership abilities due to the support they receive from the community. Additionally, for individuals who struggle with public speaking, being part of the congregation provides an opportunity to practice and improve these skills. As an illustration, Sara, a 47-year-old member from Huehuetenango, shared the following perspective:

At the church, participation is encouraged which helps to alleviate any anxiety one may feel. Even if our vocabulary is limited, we are still able to express ourselves. The community council holds meetings to promote unity within the church and provide support for everyone to continue walking in God’s path.

During our conversation, Sofia brought up how religious groups encourage young people to form groups and collaborate to foster growth. She shared her belief that this is an important aspect of being part of a religious community. She expressed: “It is also on behalf of the Church that when the young people, the religious leaders, encourage them [youth]. Therefore, they have a group, that they keep going, that these are the good things.”

Darvin emphasized the importance of taking responsibility and collaborating with the community. He also suggested that getting involved with the church could increase participation and engagement. Darvin stated: “Well if you see that here they say that you should be responsible, that you agree to work with the community or in the church, that you be a good leader so that you can continue.”

### Maya-Mam Indigenous competency: nurturing relationship

During conversations with participants, it was emphasized that community leaders have the responsibility to support and care for their followers. They are expected to not only provide resources and opportunities for individuals to better their lives but also to safeguard them from potential risks.

By taking on this protective role, leaders establish a sense of trust and security within the community. They become champions for the well-being of their followers, promoting a nurturing and supportive environment where individuals can grow and thrive. This responsibility highlights the crucial role leaders play in advancing the community’s development and ensuring the overall welfare and protection of its members.

Rodolfo shared his belief that leadership means remaining aware of the community, serving it, and prioritizing the needs of the people. He mentioned: “Leadership is about serving the community, and being attentive to the community itself because good leadership remembers the people, to serve them. Leadership means receiving feedback to know that the community is doing the right thing.”

Beatriz explained that certain leaders have been working on initiatives aimed at reducing undernourishment in the area. She explained: “Because from time to time they talk, for example, they ask for children who are malnourished. There they should be told to send them help from the municipality.”

Celeste advocated for ensuring that the community abides by the law, while also emphasizing the importance of the leader’s ability to guide members in case of a newborn certificate. She stated: “To serve the community. Matters of whatever concern the legislatures. Some dilemmas among the members of the community, a problem, or a birth certificate. It’s a lot of things.”

Amapola has taken on more responsibilities, which reflects her willingness to contribute and her value of being responsible with her time. However, she is not interested in taking on all the leadership roles. As she mentioned:

It’s important to acknowledge people’s positivity and their ability to invest their time in serving their community. Sometimes, people are re-elected based on the perception that they can juggle multiple responsibilities. However, it’s crucial to recognize that time is a finite resource and it’s important to be honest about what one can realistically accomplish. So, I feel that I also must see the time be positive and be honest.

### Maya-Mam Indigenous competency: equal opportunities

Participants expressed a strong desire for leaders who embrace inclusivity and ensure the involvement of all community members. They emphasized the importance of this value because, through developmental programs and increased access to information, individuals have become more aware of their rights and responsibilities.

Inclusivity in leadership means that all community members, regardless of background or status, have a voice and are actively engaged in decision-making. By prioritizing inclusivity, leaders foster a sense of unity, fairness, and equality within the community. They create an environment where everyone feels valued, respected, and empowered to actively participate in shaping the community’s future.

As a leader of an organization, Lorenza believes it is important to ensure that no one is excluded and to strive for cooperation, assistance, and collaboration with the community. She emphasizes that inclusivity is a key aspect of leadership. She explained:

As a leader, it’s important to acknowledge that being part of an organization means decisions cannot always be made solely on personal desires. Sometimes, we may take our opinions too seriously and exclude others, which goes against good leadership practices. Accessibility is key – a true leader is approachable and willing to collaborate and support, rather than solely commanding and controlling.

Sara argued that women should be given equal opportunities to lead. She explained that women possess the same rights and abilities as men to lead communities. She expressed: “Let us say no, not only men have the right, but also women. We can promote a community or something like that.”

Waleska, a 19-year-old resident of Huehuetenango, noted that while everyone in the community has the right to vote, it is rare to see women in leadership positions. She acknowledged that discriminatory bylaws and practices often make women feel excluded and expressed a desire for someone to break the glass ceiling. She explained:

Here everyone can vote, but it is not very common for women to be leaders of the community. Sometimes the laws, sometimes the assembly votes for men rather than women. They are choosing more people who are men.

During a conversation with Eunice, a 57-year-old resident of Huehuetenango, she emphasized the importance of having a leader who values community participation and collaborates with the community assembly. All members of the community should have a say in the decision-making processes. This is crucial for effective leadership. She expressed:

The leader is the one who must be on par with everyone, not to go on stage and say what he thinks, but to have proposals together with the assembly. The leader is the one who must know how to say everything that he thinks and then he can communicate with other excluded members because it is like leading together with the assembly. Not feel bigger or smaller, because he does not have to be in a main chair, but rather he has to be on a par with everyone.

### Maya-Mam Indigenous competency: transparency

Community members emphasized the crucial role of honesty in leadership, driven by negative experiences in the past that have caused a sense of mistrust (International Labor Office, 2021). These experiences have demonstrated the damaging effects of dishonesty on the community and highlighted the need for leaders who prioritize integrity and transparency.

Participants value leaders who are truthful and open in their actions, words, and intentions. They recognize that dishonesty can create divisions and erode trust, and therefore emphasize the significance of consistent honesty in leaders. Lorenza mentioned that being truthful also requires acknowledging your commitment when proposing a program and serving the community out of love. She expressed:

What I would look for from the leader is they are honest because an honest person, we are going to support. Also dedicated because there are people who are honest but no, do not give their time. So, there are people who say that they accept to serve, but behind, they are looking to see what is left of that payment to grab. So that kind of person would not lead our community.

According to Julieta, the chosen leader values honesty in his work, communicates clearly about what is happening, and holds those who engage in illegal actions accountable. She explained:

We vote for the motivation he has. The community and the assembly trust that person, we are so clear about what he did in his role as president since he had noticed all the dirty things within the community, and through him, all the corruption problems came to light. So, there you see the person with that capacity to deliver and denounce the things that are hidden. He and his team worked because thank God it was possible to discover the things that were hidden there because everything came to light. He even had threats, but the assembly itself defended him and spoke for him. So that’s why he is president right now.

## Discussion

Following recommendations by Salmon et al. (2023), this study adopted a leadership competency model as an organizing framework to communicate findings more effectively for a broad range of researchers. This study provides valuable insight into the leadership competencies of the Indigenous Maya-Mam community. It aligns with previous research that emphasizes the importance of analyzing leaders’ skills, knowledge, values, traits, and motives (Boyatzis, 1982; Čukanović-Karavidić et al., 2016; Hornby and Thomas, 1989; Jacobs, 1989; McClelland, 1973). By adopting the Maya-Mam leadership competency model, the study sheds light on the specific competencies demonstrated by Maya-Mam leaders, which is significant due to the limited information available on Maya-Mam Indigenous groups and their leadership practices (Ciborowski et al., 2021; Ekern, 2011).

In this research, an essential competency that emerged was the notion of leading by example, which holds great significance due to the participants’ dissatisfaction with leaders who engage in demagoguery and fail to follow through on their promises. The participants emphasized the role of leaders as public figures who have the power to inspire and motivate community members to actively contribute to the progress of the community. Furthermore, the participants highlighted the significance of evaluating potential leaders based on their capacity to guide and govern not only at the community level but also within their own families. This suggests that community members take into consideration a leader’s past experiences and contributions, as well as their performance in previous positions when making decisions regarding leadership selection. Similar findings information was found in Avalos-Pelaez’s (2017) research, where she expressed that the leaders in Maya communities based their leadership on showing their examples and being judged by followers.

The ability to speak both Mam and Spanish is greatly appreciated by Indigenous leaders in the Mams community. This bilingual competency allows them to effectively communicate the community’s needs in Spanish, which is the dominant language in broader society. However, language barriers often prevent the Mams people from accessing resources and services. International Labor Office (2021) also reported similar findings, stating that Indigenous women view learning Spanish as a crucial step towards improving their self-esteem. By raising awareness of their potential, they are better equipped to assess their opportunities (Caxaj and Kolol Qnan Tx'otx' Parroquia de San Miguel Ixtahuacan, 2018; Caxaj et al., 2014).

Although the research findings are specific to this Maya-Mam Indigenous group and cannot be applied to all Maya-Mam Indigenous communities, they contribute to the growing body of research on Indigenous leadership in non-Western contexts (Zhang et al., 2012). To build on this work, future research could replicate and adapt the leadership competency model in other Indigenous contexts. Program implementers working with Maya-Mam communities could also utilize the model to identify potential leaders who possess the skills and competencies identified in this research. This may facilitate the implementation of effective programs.

The researchers recognized that the Maya-Mam were located in different areas, which could have influenced our findings. However, understanding a group fully requires expanding research to the locations in which they live and work. Another limitation was the language barrier between the principal researcher and the interviewees could have taken place. However, it was addressed this issue by having a native speaker facilitate in situations where a common language was not shared.

The Maya-Mam leadership competency model sheds light on the specific skills demonstrated by Maya-Mam leaders, offering valuable insights into the leadership development of the Indigenous Maya-Mam community. This study is significant because it addresses the limited information available on the Maya-Mam Indigenous groups and their leadership practices. The key findings of the study include the importance of leading by example, the role of leaders as public figures inspiring community members, the significance of inclusive leadership and equal opportunities, the value of bilingualism in effective communication, and the collective nature of the Indigenous community. These insights contribute to the growing body of research on Indigenous leadership and offer practical applications for program implementers in Maya-Mam communities.

## Data Availability

The raw data supporting the conclusions of this article will be made available by the authors, without undue reservation.
