# Supplementary material for: Indigenous Maya-Mam leadership competencies: a grounded theory study
Source: Front Sociol. 2025 Jan 10;9:1463562. doi: 10.3389/fsoc.2024.1463562 (PMC11757294; doi:10.3389/fsoc.2024.1463562)
Supplement: Supplementary file 1 [file Data_Sheet_1.docx]

**INTERVIEW**

I would like to discuss indigenous leadership in Guatemala. I understand that some of the questions might be sensitive to discuss, but I would like to gain a better understanding of what is happening in this significant process. Please be assured that everything you share with me will remain confidential; no one will know the specifics of what you tell me. This conversation is a safe space. With your consent, may I record this conversation?

Instructions: Before we begin the interview, please provide the following information:

Start recording the interview.

Pseudonym of the interviewee:

Age:

Position in the community:

1. Can you tell me a little about yourself?
   1. **Follow-up:** How long have you been living in the community?
2. Have you heard of the term "leadership"?
   1. **Follow-up:** If yes, what have you heard about it?
   2. **Follow-up:** Who is the person responsible for organizing and guiding the group during community assemblies?
   3. **Follow-up:** Why do they assume this role?
3. If you had to choose a person to lead the community, what attributes or characteristics would you consider?
   1. **Follow-up:** What are the reasons behind choosing that person?
4. Are you familiar with the role of the auxiliary mayor in your community?
   1. **Follow-up:** What are the responsibilities of the assistant mayor?
   2. **Follow-up:** What are the reasons behind choosing that person?
   3. **Follow-up:** Can you describe the process of selecting the assistant mayor?
   4. **Follow-up:** What are the requirements to become an assistant mayor?
   5. **Follow-up:** Can women hold the position of auxiliary mayor?
   6. **Follow-up:** Please provide an example if you know of any.
5. Have you heard about the president of COCODE (Community Development Council)?
   1. **Follow-up:** What is the role of the president?
   2. **Follow-up:** What are the reasons behind choosing that person?
   3. **Follow-up:** Can you describe the process of selecting the president?
   4. **Follow-up:** What are the requirements to become a president?
   5. **Follow-up:** Can women be presidents of COCODE?
   6. Please share an example if you know of any.
6. What are the differences between the COCODE president and the auxiliary mayor?
7. How does the leader/assistant mayor communicate with the community?
8. In general, what roles do women play in the community?
9. How are women organized within the community?
   1. **Follow-up:** Who is responsible for organizing the meetings?
   2. **Follow-up:** Why do they organize these meetings?
   3. **Follow-up:** Who tends to be the most vocal during these meetings?
   4. **Follow-up:** Why do you think those individuals are the most vocal?
10. What is the role of youth in the community?
    1. **Follow-up:** How are young people organized?
    2. **Follow-up:** Do young people have a voice in community meetings?
    3. **Follow-up:** If yes, why is their input valued?
11. If you were chosen as a leader, would you accept the position?
    1. **Follow-up:** What are the reasons behind your decision to accept or decline the position?
12. Are you familiar with the concept of the baton?
    1. **Follow-up:** What does it symbolize?
13. Do you know how the community leadership election system is managed?
